# Interaction between Omeprazole and Gliclazide in Relation to CYP2C19 Phenotype

**DOI:** 10.3390/jpm11050367

**Published:** 2021-05-03

**Authors:** Tanja Dujic, Sandra Cvijic, Amar Elezovic, Tamer Bego, Selma Imamovic Kadric, Maja Malenica, Alisa Elezovic, Ewan R. Pearson, Aida Kulo

**Affiliations:** 1 Department of Biochemistry & Clinical Analysis, Faculty of Pharmacy, University of Sarajevo, 71000 Sarajevo, Bosnia and Herzegovina; tamer.bego@ffsa.unsa.ba (T.B.); selma.imamovic@ffsa.unsa.ba (S.I.K.); maja.malenica@ffsa.unsa.ba (M.M.); 2 Department of Pharmaceutical Technology and Cosmetology, University of Belgrade-Faculty of Pharmacy, 11221 Belgrade, Serbia; gsandra@pharmacy.bg.ac.rs; 3 Control Laboratory, Agency for Medicines and Medical Devices of Bosnia and Herzegovina, 71000 Sarajevo, Bosnia and Herzegovina; a.elezovic@almbih.gov.ba; 4 Department of Pharmaceutical Technology, Faculty of Pharmacy, University of Sarajevo, 71000 Sarajevo, Bosnia and Herzegovina; alisa.elezovic@ffsa.unsa.ba; 5 Division of Population Health & Genomics, School of Medicine, University of Dundee, Dundee DD1 9SY, Scotland, UK; e.z.pearson@dundee.ac.uk; 6 Department of Pharmacology, Clinical Pharmacology and Toxicology, Faculty of Medicine, University of Sarajevo, 71000 Sarajevo, Bosnia and Herzegovina; aida.kulo@mf.unsa.ba

**Keywords:** omeprazole, gliclazide, CYP2C19, physiologically based pharmacokinetic modeling, drug–drug interaction, drug–drug–gene interaction, type 2 diabetes, adverse drug reaction, hypoglycemia

## Abstract

The antidiabetic drug gliclazide is partly metabolized by CYP2C19, the main enzyme involved in omeprazole metabolism. The aim of the study was to explore the interaction between omeprazole and gliclazide in relation to CYP2C19 phenotype using physiologically based pharmacokinetic (PBPK) modeling approach. Developed PBPK models were verified using in vivo pharmacokinetic profiles obtained from a clinical trial on omeprazole-gliclazide interaction in healthy volunteers, CYP2C19 normal/rapid/ultrarapid metabolizers (NM/RM/UM). In addition, the association of omeprazole cotreatment with gliclazide-induced hypoglycemia was explored in 267 patients with type 2 diabetes (T2D) from the GoDARTS cohort, Scotland. The PBPK simulations predicted 1.4–1.6-fold higher gliclazide area under the curve (AUC) after 5-day treatment with 20 mg omeprazole in all CYP2C19 phenotype groups except in poor metabolizers. The predicted gliclazide AUC increased 2.1 and 2.5-fold in intermediate metabolizers, and 2.6- and 3.8-fold in NM/RM/UM group, after simulated 20-day dosing with 40 mg omeprazole once and twice daily, respectively. The predicted results were corroborated by findings in patients with T2D which demonstrated 3.3-fold higher odds of severe gliclazide-induced hypoglycemia in NM/RM/UM patients concomitantly treated with omeprazole. Our results indicate that omeprazole may increase exposure to gliclazide and thus increase the risk of gliclazide-associated hypoglycemia in the majority of patients.

## 1. Introduction

After metformin, sulfonylureas (SUs) are the most commonly prescribed drugs in the treatment of type 2 diabetes (T2D) worldwide [1]. This is due to their high efficacy and least cost, despite the development of newer antidiabetic agents. SUs are particularly widely used in low and middle-income countries, where they are recommended as the treatment of choice for T2D when metformin monotherapy fails to achieve glycemic targets [2].

Hypoglycemia is the most common and potentially most serious side effect of sulfonylurea treatment [3]. Severe hypoglycemia is associated with significantly increased morbidity and mortality [4,5,6]. Among different SUs, gliclazide is recommended as the treatment of choice in many countries, as it seems to be associated with a lower risk of hypoglycemia and lower morbidity and mortality compared with other SUs [7,8]. Furthermore, gliclazide has been included in the WHO Model List of Essential Medicines, based on its safety data in elderly patients. However, in a large-population-based cohort study, gliclazide showed a similar risk of hypoglycemia compared with other SUs [9].

Gliclazide is extensively metabolized in the liver to inactive metabolites. Although CYP2C9 enzyme is involved in its metabolism [10], gliclazide pharmacokinetics (PK) seem to be affected by *CYP2C19* genetic polymorphisms [11,12]. Observed gliclazide AUC was 3.4-fold [11] and 5.1-fold [12] higher in CYP2C19 poor metabolizers (PM) compared to normal metabolizers (NM) following single oral administration of gliclazide in healthy Chinese volunteers.

Gastrointestinal (GI) problems, such as gastroesophageal reflux disease, are common in patients with T2D [13]. The prevalence of GI symptoms in those patients could be as high as 40% [13]. Considering that about 70% of patients with diabetes use oral hypoglycemic agents, millions of individuals are exposed to concomitant use of oral antidiabetic and acid-suppressing medications [14]. Proton pump inhibitors (PPIs) are the treatment of choice for gastroesophageal reflux disease and are primarily metabolized by CYP2C19 [15]. The potential for drug-drug interactions (DDI) is highest for omeprazole and its stereo-isomer esomeprazole, both metabolized almost entirely by CYP2C19 [15,16]. Furthermore, the impact of PPIs on the PK, pharmacodynamics, and therapeutic response of drugs metabolized by CYP2C19 was shown to depend on *CYP2C19* genotype, with the highest effect seen in CYP2C19 NM [17,18] and ultrarapid metabolizers (UM) [19].

Physiologically based pharmacokinetic (PBPK) modeling is well established in the pharmaceutical industry and is accepted by regulatory agencies for the prediction of DDIs of new drugs [20,21,22]. It has been widely used especially for CYP-mediated DDIs [23]. Considering the possibility of interaction via CYP2C19, we aimed to explore the impact of omeprazole on gliclazide PK, in relation to CYP2C19 phenotype using a PBPK approach. Developed PBPK models were validated using in vivo PK profiles obtained in a clinical trial on omeprazole-gliclazide interaction in healthy volunteers, CYP2C19 normal/rapid/ultrarapid metabolizers (NM/RM/UM). Finally, the possible interaction between the two drugs was explored in the cohort of patients with T2D treated with gliclazide.

## 2. Materials and Methods

### 2.1. PBPK Modeling and Simulations

The PBPK models were constructed using Simcyp Simulator (version 19.1, Certara UK Ltd., Sheffield, UK). Clinical data from the literature were used to verify the models for each drug. Graphical data of the published mean plasma concentration-time profiles were digitized using GetData Graph Digitizer (version 2.26, S. Fedorov). Simcyp default values for ‘Sim-Healthy Volunteers’ were used for healthy Caucasian population, except for phenotype frequencies of CYP2C9 and CYP2C19 enzymes, and their abundances in the liver. Considering that both enzymes are highly polymorphic, the latest published frequencies for the European population were used. For CYP2C9, the following frequencies were entered: 0.629 for NM (extensive metabolizer, EM), 0.345 for intermediate metaboliser (IM), and 0.026 for PM phenotype [24]. For CYP2C19, phenotype frequencies for the European population were: 0.319 for RM/UM (0.047 for UM and 0.272 for RM), 0.396 for NM (EM), 0.261 for IM and 0.025 for PM phenotype [25]. In Simcyp, differences in CYP activities between phenotypes are accounted for using functional enzyme abundances (pmol of enzyme/mg of microsomal protein). For CYP2C9, we used Simcyp default liver abundance values for EM individuals, whereas abundances for other phenotypes were adjusted based on the estimated enzyme activities compared to the EM phenotype. Thus, liver abundance for CYP2C9 EM was set to 73, for IM to 47.6 and for PM phenotype to 13.7 pmol/mg protein (corresponding to activity scores of 2.0, 1.0–1.5, and 0–0.5 for NM, IM and PM phenotype, respectively) [24]. For CYP2C19, we used Simcyp default liver abundance values for EM and UM phenotypes, 4.4 and 8.7 pmol/mg protein, respectively, and a mean value of 6.6 pmol/mg for the RM phenotype. For IM phenotype abundance was set to 1.32, and for PM to 0.022 pmol/mg (corresponding to activity scores of 0.30 and 0.005 for IM and PM compared to EM phenotype, respectively) [26].

#### 2.1.1. Omeprazole Model Construction and Validation

The minimal PBPK model for omeprazole was developed based on previously published models [27,28]. The input parameters are given in Table 1. Absorption was described by a first-order absorption model in line with previous studies [27,28]. The steady-state volume of distribution (V_ss_) value was based on previous models [28,29]. The intrinsic clearance values per pmol of enzymes were back calculated from the clinical data after oral administration of a single dose of omeprazole in subjects with a functional CYP2C19 [30], taking into account the estimated contributions of CYPs to the metabolism of omeprazole (f_m_) in CYP2C19 NM/RM/UM metabolizers [31]. As omeprazole is also a mechanism-based inhibitor of CYP2C19, the values for the inhibitor concentration that supports half the maximal rate of inactivation (K_app_) and maximal rate of enzyme inactivation (k_inact_), were optimized to fit the observed concentration-time profiles after single and multiple oral dosing of 20 mg and 40 mg omeprazole.

For model validation, clinical PK data from the literature following single and multiple oral administration of 20 mg and 40 mg omeprazole were used [30]. The simulations were performed using 10 virtual trials with 10 individuals in each trial, matched for age, gender, body weight and CYP2C19 phenotype with clinical trial subjects [30].

#### 2.1.2. Gliclazide Model Construction and Validation

The full PBPK model was developed for gliclazide using a middle-out approach. The input parameter values are shown in Table 2. The V_ss_ was predicted by Simcyp Rodgers et al. + ion membrane permeability model. The Kp scalar of 3.5 was used to adjust the predicted V_ss_ value to those observed in humans in vivo (0.19–0.46 L/kg) [32,33,34,35]. The intrinsic clearances per pmol of CYP enzymes were calculated using total intrinsic clearance measured in vitro in human liver microsomes [10], and f_m_ values obtained from in vivo data [36]. The drug clearance based only on enzyme kinetics data was underpredicted around 3-fold in comparison to the in vivo i.v. data [35], indicating that active drug uptake into the liver should also be considered. As a recent study showed that gliclazide is a substrate of hepatic OATP1B1 transporter [37], a permeability limited liver model was used and in vitro values for Michaelis–Menten constant (K_m_) and maximum transport rate (J_max_) obtained in human embryonic kidney (HEK293T) cells stably expressing OATP1B1, were added as inputs to simulate active drug uptake into the liver. Considering the difference in abundance and activity of the transporter between liver and the transfected cell line, a relative activity factor (RAF) was optimized to fit the observed clinical data. Liver passive diffusion clearance (Cl_PD_) of gliclazide was estimated [38] based on experimental logD_7.4_ value [39].

The Simcyp Advanced Dissolution, Absorption and Metabolism (ADAM) model was used to simulate gliclazide absorption from the GI tract. For oral immediate-release (IR) formulations, the Simcyp Diffusion Layer Model was used to predict drug dissolution based on the published experimentally obtained pH-dependent solubility data [40]. For modified-release (MR) formulations, our experimental dissolution profiles were used. Since the previous study suggested that gliclazide is a substrate for the efflux Mrp2 transporter in rats [41], simulation of the effect of intestinal MRP2 was included in the model. Value for MRP2-mediated intrinsic clearance (Cl_int,T_) was optimized to fit the predicted PK profiles with the observed clinical data after oral drug dosing.

**Table 1:** Input parameters for omeprazole physiologically based pharmacokinetic (PBPK) model.

Parameter	Value	Method/Reference
Physicochemical properties		
Molecular weight (g/mol)	345.4	
Log P	2.23	Ogilvie et al., 2011 [27]
pKa	4.4; 8.7	Ogilvie et al., 2011 [27]
Compound type	Ampholyte	Ogilvie et al., 2011 [27]
B/P	0.59	Ogilvie et al., 2011 [27]
fu	0.043	Ogilvie et al., 2011 [27]
Absorption		
Absorption model	First-order	
fa	1	Ogilvie et al., 2011 [27]
ka (1/h)	6	Ogilvie et al., 2011 [27]
Q_gut_ (L/h)	18.6	Predicted by Simcyp
fu_gut_	0.043	Equal to fu (assumed)
Distribution		
Distribution model	Minimal PBPK model	
V_ss_ (L/kg)	0.15	Deng 2016 [29], Wu et al., 2014 [28]
Elimination		
Cl_R_ (L/h)	0.037	Wu et al., 2014 [28], Feng et al., 2015 [42]
f_m_ CYP2C19 (%)	86	Literature data (a)
f_m_ CYP3A4 (%)	14	Literature data (a)
Cl_int_ CYP2C19 (µL/min/pmol)	75.1	Calculated (b)
Cl_int_ CYP3A4 (µL/min/pmol)	0.39	Calculated (b)
Interaction		
Ki CYP2C19 (µM)	5	Ogilvie et al., 2011 [27]
fu_mic_	1	Ogilvie et al., 2011 [27]
K_app_ CYP2C19 (µM)	0.3	Optimized (c)
k_inact_ CYP2C19 (1/h)	5.5	Optimized (c)
fu_mic_	1	Simcyp default

Log P, octanol/water partition ratio; pKa, dissociation constant; B/P, blood/plasma partition ratio; fu, fraction unbound; fa, fraction absorbed; ka, absorption rate constant; Q_gut_, gut flow; fu_gut_, fraction unbound in enterocytes; V_ss_, volume of distribution at steady state; CL_R_, renal clearance; f_m_, fraction metabolized; CL_int_, in vitro intrinsic clearance; Ki, inhibitor concentration that supports half maximal inhibition; fu_mic_, fraction unbound in vitro; K_app_, inhibitor concentration that supports half the maximal rate of inactivation; k_inact_, maximal rate of enzyme inactivation; fg, fraction escaping gut metabolism; (a) average value for CYP2C19 EM/RM/UM phenotypes [31]; (b) retrograde calculated value based on observed Cl_po_ after 20 mg single dose of omeprazole (L/h) [30] assuming fa * fg = 1 [28,43]; (c) optimized based on values for enantiomers [28].

The model was validated using clinical PK data from published trials in Caucasians obtained after i.v. infusion of 30 mg gliclazide [35], single oral administration of the 80 mg gliclazide IR tablet [44], and single oral administration of the 30 mg gliclazide MR tablet [35]. The simulations were performed using 10 virtual trials with 10 individuals in each trial, matched for age, gender and body weight with respective clinical trial volunteers.

**Table 2:** Input parameters for gliclazide PBPK model.

Parameter	Value	Method/Reference
Physicochemical properties		
Molecular weight (g/mol)	323.4	
Log P	2.04	El-Sabawi, et al., 2014 [45]
pKa	2.9; 5.8	Grbic et al., 2011 [40]
Compound type	Ampholyte	Grbic et al., 2011 [40]
B/P	0.68	Predicted by PK-Sim
fu	0.15	Proks et al., 2018 [46]
Absorption		
Absorption model	ADAM	
Human jejunal effective permeability, P_eff,man_ (10^−4^ cm/s)	3.68	Grbic et al., 2011 [40]
Solubility	pH-profile entered	Grbic et al., 2011 [40]
Immediate Release Formulation	Diffusion layer model for dissolution	
Modified Release Formulation	Experimental dissolution profile entered	
Distribution		
Distribution model	Full PBPK model	
V_ss_ (L/kg)	0.306	Predicted by Simcyp (a)
Kp scalar	3.5	Estimated (b)
Elimination		
f_m_ CYP2C19 (%)	76	Tod et al., 2013 [36]
f_m_ CYP2C9 (%)	24	Tod et al., 2013 [36]
Cl_int_ CYP2C19 (µL/min/pmol)	0.273	Calculated (c)
Cl_int_ CYP2C9 (µL/min/pmol)	0.004	Calculated (c)
fu_mic_	0.82	Predicted by Simcyp
Transport		
Intestinal MRP2 Cl_int,T_ (µL/min)	5.0	Optimized
Liver passive diffusion clearance Cl_PD_ (mL/min/10^6^ hepatocytes)	7.95 × 10^−4^	Calculated (d)
OATP1B1 K_m_ (µM)	30.2	Chen et al., 2018 [37]
OATP1B1 J_max_ (pmol/min/10^6^ cells)	12.9	Chen et al., 2018 [37]
OATP1B1 RAF	5.75	Optimized

Log P, octanol/water partition ratio; pKa, dissociation constant; B/P, blood/plasma partition ratio; fu, fraction unbound; V_ss_, volume of distribution at steady state; Kp scalar, scalar applied to all predicted tissue/plasma partition coefficients; f_m_, fraction metabolized; CL_int_, in vitro intrinsic clearance; fu_mic_, fraction unbound in the in vitro microsomal incubations; Cl_int,T_, in vitro transporter-mediated intrinsic clearance; Cl_PD_, liver passive diffusion clearance; K_m_, Michaelis–Menten constant; J_max_, maximum transport rate in vitro; RAF, relative activity factor; (a) method by Rodgers et al. + ion membrane permeability; (b) estimated by matching the predicted V_ss_ value to the in vivo observed V_ss_ values (0.19–0.46 L/kg); (c) calculated based on the microsomal total intrinsic clearance [10], CYP abundances in the liver and the f_m_ values [36]; (d) Calculated [38] based on logD_7.4_ value [39].

#### 2.1.3. Simulation of Omeprazole-Gliclazide Interaction

We simulated the DDI study between omeprazole and gliclazide according to our clinical trial protocol. The simulations were run for 192 h, using 10 virtual trials with 10 individuals in each virtual trial, matched for age, gender, body weight, and CYP2C9, CYP2C19, and OATP1B1 phenotypes with the real trial volunteers. Virtual individuals received 20 mg of oral omeprazole for 5 days, and an oral dose of 40 mg gliclazide was co-administered on day 5.

In the next step, we performed simulations of DDI in virtual individuals who received 20 mg omeprazole for 5 days and 80 mg gliclazide IR tablet on day 5, for NM, RM/UM, IM and PM phenotypes separately. Finally, we simulated real-world multiple dosing scenarios with NM/RM/UM and IM individuals receiving daily 40 or 80 mg omeprazole concomitantly with 80 mg gliclazide for 20 days.

#### 2.1.4. Assessment of PBPK Models Performance 

The performance of the models was assessed by visual inspection of the plasma concentration-time profiles and by comparison of the predicted PK parameters to the clinically observed data (C_max_, t_max_, AUC). Observed data from the literature were reported as arithmetic means and standard deviations, geometric means with 95% confidence intervals (CIs) or medians with range. For comparison with the simulated values, arithmetic means were transformed to geometric means and 95% CIs, if these were not reported [47]. The fold error was calculated as the ratio of simulated and observed parameter values. The predicted values within 2-fold range of observed data were considered acceptable.

For DDI evaluation, geometric mean ratios of predicted gliclazide C_max_ and AUC_0–∞_ in the presence/absence of omeprazole were determined as follows:

DDI AUC ratio = AUC_0–∞_ of gliclazide co-administrated with omeprazole/AUC_0–∞_ of gliclazide administered alone (control).

DDI C_max_ ratio = C_max_ of gliclazide co-administrated with omeprazole/C_max_ of gliclazide administered alone (control).

The 90% CI range was presented for DDI ratios in line with guidelines for drug interaction studies [20].

### 2.2. Clinical Trial

#### 2.2.1. Subjects

The study protocol was approved by the Ethics Committee of the General Hospital “Prim. Dr. Abdulah Nakas” (100-78/18), and by the Ethics Committee of the Faculty of Medicine, University of Sarajevo (02-3-4-8798/18), Sarajevo, Bosnia and Herzegovina (B&H). All procedures were conducted in line with the Good Clinical Practice and the Declaration of Helsinki. Prior to inclusion in the study, the nature and purpose of the study were explained, in both written and verbal form, to each volunteer who then gave the informed written consent to participate in the study. The study was registered at the ClinicalTrials.gov (ID NCT04198948).

Fifteen healthy volunteers (men, age 18–30 years, non-smokers) with CYP2C19 NM/RM/UM status were recruited. The exclusion criteria included: a medical history of hepatic, renal, gastrointestinal or hematologic disease or any acute or chronic disease; drug allergy to sulfonylureas or PPIs; history of drug abuse; abnormalities in physical examination, ECG or routine clinical laboratory tests (including fasting blood glucose concentration); medication use during the 14 days prior to and during the study period; grapefruit, grapefruit juice, alcohol, beverages or food containing methylxanthines use during the 72 h prior to and during the study period.

#### 2.2.2. Study Design

A randomized, placebo controlled, two-sequence, two-period crossover study, with a 10-day washout between the study periods, was performed to compare the effects of gliclazide (40 mg, half of an 80 mg tablet of Diprian^®^, Hemofarm d.o.o. Banja Luka, B&H) combined with placebo with those of gliclazide combined with omeprazole (20 mg, Ulcosan^®^, Bosnalijek d.d. Sarajevo, B&H). Prior to administration of gliclazide, omeprazole or placebo was administered once daily at 8 AM for 4 days (on day 1 until day 4). On day 5 of each admission period, after an overnight fast, the volunteers were admitted to the hospital. After the blood glucose level was measured using a glucometer, a single dose of 40 mg gliclazide with 240 mL of water was co-administered with either omeprazole or placebo, according to their treatment assignment. Drugs were given in a sitting position, at 8 AM. Volunteers were not permitted to lie down or sleep for the next 4 h after taking drugs, as well as to perform any strenuous activities for the next 24 h. In order to counteract the blood glucose-lowering action of gliclazide, volunteers received standardized meals: breakfast at 30 min after gliclazide intake, snacks after 2 and 3 h, a lunch (warm meal) after 4 h, a snack after 6 h, dinner after 8 h, and additional snacks at 10 h and 12 h after drugs administration. No other food and drink (with the exception of water) were allowed. For safety reasons, the volunteers were under direct medical supervision throughout the 24 h following drugs administration. Fast-acting carbohydrates, glucose solution for i.v. use and glucagon for intramuscular use were available in case of hypoglycemia.

#### 2.2.3. Sampling

Venous blood samples were collected on day 5 of each admission period before dosing (0 h) and 0.5, 1, 1.5, 2, 2.5, 3, 3.5, 4, 4.5, 5, 6, 8, 10, 12, 15 and 24 h after gliclazide administration. Serum samples for gliclazide quantification were separated within 30 min following each blood sample collection and stored at −80 °C until the analysis.

#### 2.2.4. Quantification of Gliclazide

Serum gliclazide concentrations were quantified using a validated stable isotope dilution liquid chromatography-tandem mass spectrometry (LC-MS/MS) method. In brief, a total of 10 µL serum was added to 90 µL acetonitrile containing 100 ng/mL of d4-gliclazide and 0.1% formic acid. After 5 s vortex and centrifugation at 16,110× *g* for 10 min (4 °C), the supernatant was subjected to LC-MS/MS analysis using a Dionex U3000 LC system connected to a Thermo Quantum Ultra mass spectrometer with an IonMax interface (all instruments were from Thermo Scientific, Hemel Hempstead, United Kingdom). A volume of 5 µL supernatant was injected by the LC system and the analytes were separated on an Inertsil HILIC column (5 μm, 150 × 2.1 mm (Supelco)) using an isocratic mode at a flow rate of 0.65 mL/min. Gliclazide was quantitated by the triple quadrupole mass spectrometer in ESI positive ion mode. The targeted ion transitions (m/z) for gliclazide and d4-gliclazide were 324–>127 and 328–>127, respectively. The ion spray voltage was set at 4500 V, capillary temperature at 275 °C, acquisition time at 100 ms, the resolution of both quadrupoles at 0.7, collision gas pressure (argon) at 1.5 mTorr, optimized collision induced dissociation energy at 38 eV and tube lens voltage at 40 V. Calibration curves and quality control samples were included in each batch of analysis. The intra- and inter-assay imprecision (%CV) for gliclazide were 4 to 8% and 4 to 5%, respectively. The intra- and inter-assay accuracy for gliclazide ranged from −13 to 1% and from −8 to −3%, respectively. The limit of quantification was 3.1 ng/mL.

#### 2.2.5. Pharmacokinetic Analysis

PK parameters were calculated in a noncompartmental analysis using PKSolver, a program for PK data analysis in Microsoft Excel. The area under the plasma gliclazide concentration-time curve (AUC) up to the last concentration measured (AUC_0–t_) was determined using the linear trapezoidal rule.

#### 2.2.6. Genotyping

Genomic DNA was extracted from blood samples by QIAamp DNA Blood Midi Kit (Qiagen, Hilden, Germany). Polymorphisms in the *CYP2C19* gene: CYP2C19*2 (rs4244285) and CYP2C19*17 (rs12248560), *CYP2C9* gene: CYP2C9*2 (rs1799853), CYP2C9*3 (rs1057910), and c.521T>C (rs4149056) variant in the *SLCO1B1* gene were genotyped by specific TaqMan Drug Metabolism SNP genotyping assays (Applied Biosystems, Foster City, CA, USA).

The following CYP2C19 phenotypes were assigned based on diplotypes: NM (*1/*1), RM (*1/*17), UM (*17/*17), IM (*1/*2, *2/*17) and PM (*2/*2) [25]. All volunteers were non-carriers of the CYP2C19*2 allele and thus NM, RM or UM metabolizers. For CYP2C9 assigned phenotypes were: NM (*1/*1), IM (*1/*2, *1/*3, *2/*2) and PM (*2/*3, *3/*3) [24]. For OATP1B1 (*SLCO1B1* gene), the following phenotypes were assigned based on the rs4149056 genotype: normal transporter (c.521TT), intermediate transporter (c.521TC) and poor transporter (c.521CC) phenotype [48].

#### 2.2.7. Statistical Analysis

The main evaluated outcome was systemic exposure to gliclazide, expressed as AUC_0–t_. The geometric mean was calculated for AUC_0–24_. The ratio of the geometric means with 90% CIs was assessed by linear mixed models between the two treatment assignments: gliclazide and omeprazole co-administration to that of gliclazide and placebo. The obtained 90% CI was compared with the equivalence 0.8–1.25 range. The statistical analyses were performed using SAS 9.3 (SAS Institute Inc., Cary, NC, USA).

### 2.3. In Vitro Dissolution Testing

#### 2.3.1. Materials

Dissolution studies were performed with two gliclazide tablets commercially available on B&H market: half of the 80 mg Diprian^®^ tablet (tablets with the same batch number as those used in a current trial), and the 60 mg Diaprel^®^ MR tablet (Les Laboratoires Servier Industrie, Gidy, France). As the 30 mg Diamicron^®^ MR tablets administered in a published clinical study we simulated [35] were not available on B&H market, we used instead gliclazide MR formulation, the 60 mg Diaprel^®^ MR tablets, from the same manufacturer (Servier) as an approximate substitute, assuming similar dissolution profile.

Gliclazide reference standard was used for quantification (British Pharmacopoeia Chemical Reference Substance, 99.9% purity). Other used reagents were of p.a. grade. Di-potassium hydrogen phosphate and potassium hydroxide were purchased from Merck (Darmstadt, Germany), potassium dihydrogen phosphate from Fisher Chemical (Thermo Fisher Scientific, Leicestershire, UK) and 37% hydrochloric acid from Carlo Erba (Milan, Italy).

#### 2.3.2. Test Procedure

In order to estimate the drug release rate in vivo and concomitant absorption profile, dissolution tests were performed under biorelevant media-change conditions (Table 3), using USP apparatus 2 (Erweka DT 800, Heusenstamm, Germany) at 37 ± 0.5 °C and rotational speed of 100 rpm. Withdrawn aliquots were filtered, diluted and analysed for gliclazide by UV spectrophotometry (UV-1700 spectrophotometer, Shimadzu, Japan). The absorbance obtained at 226 nm was corrected by subtracting the absorbance obtained at 290 nm, according to British Pharmacopeia [49]. All measurements were performed in triplicate.

The pH values and tablet residence time in each medium were selected based on the proposed biorelevant dissolution test conditions [50,51,52]. As volunteers in the clinical trial received a breakfast 30 min after drug administration, and subsequent meals afterwards, we simulated fasted pH values for the stomach, and fed state pH values for duodenum, jejunum, ileum and colon. Medium pH 1.6 was used to simulate fasting stomach pH value, medium pH 3.0 for fasting stomach pH after 5-day treatment with omeprazole, medium pH 5.8 for fed duodenal and jejunal pH value, and medium pH 6.8 for ileal pH value. Medium pH 6.0 was used to simulate the fed state luminal conditions in the ascending colon [50].

### 2.4. Population Observational Study of Patients with T2D

We used the Scottish Ambulance Service data and the Accident and Emergency diagnosis records to identify severe cases of hypoglycemia in Tayside and Fife, Scotland. The characteristics and selection of patients who had experienced a hypoglycemic event and their respective controls were described previously [53]. From 173 cases and 826 controls who were treated with gliclazide, 48 cases and 288 controls were in the Genetics of Diabetes Audit and Research Tayside Study (GoDARTS) and had available genotype data for the two most common *CYP2C19* variants, *2 (rs4244285) and *17 (rs12248560). Logistic regression was used to analyze the effect of the *CYP2C19* variants and co-treatment with PPIs on gliclazide-induced hypoglycemia. All analyses were adjusted for known clinical predictors of hypoglycemia, including age, sex, age of T2D diagnosis, body mass index, creatinine and HbA_1c_ [53], as well as the overall number of medications used as a proxy for comorbidities [54]. The CYP2C19 phenotypes were assigned based on diplotypes in the same manner as in healthy volunteers. The analyses were performed using SAS 9.3 (SAS Institute Inc., Cary, NC, USA). Statistical significance was set at *p* < 0.05.

## 3. Results

### 3.1. PBPK Model for Omeprazole

**Figure 1:**
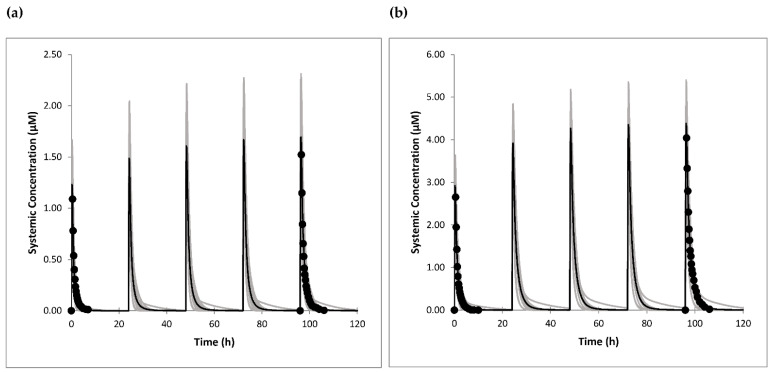
(**a**) Observed [30] (circles) and simulated (lines) plasma concentration-time profiles following multiple oral administration of 20 mg omeprazole. (**b**) Observed [30] (circles) and simulated (lines) plasma concentration-time profiles following multiple oral administration of 40 mg omeprazole. The grey lines represent means of the ten simulated trials, and the black line represents the mean of the whole virtual population.

The generated omeprazole model was used to simulate published mean plasma PK profiles obtained after single and multiple oral doses of the drug [30]. The simulated plasma concentration-time profiles after repeated dosing, once daily for 5 days with 20-mg and 40-mg omeprazole oral solutions, are presented in Figure 1a,b, respectively. The simulated profiles were in good accordance with clinically observed data. The nonlinear PK of omeprazole was well captured by optimized mechanism-based inhibition parameters. The lowest simulated percentage of active CYP2C19 levels after 5-day dosing of 40 mg omeprazole was 11% (Supplementary Figure S1). The predicted C_max_ and AUC values were within 1.1-fold of the observed data (Table 4).

### 3.2. PBPK Model for Gliclazide

As described above, a PBPK model for gliclazide was developed based on the combination of in vitro, in silico and in vivo input data (Table 2).

**Figure 2:**
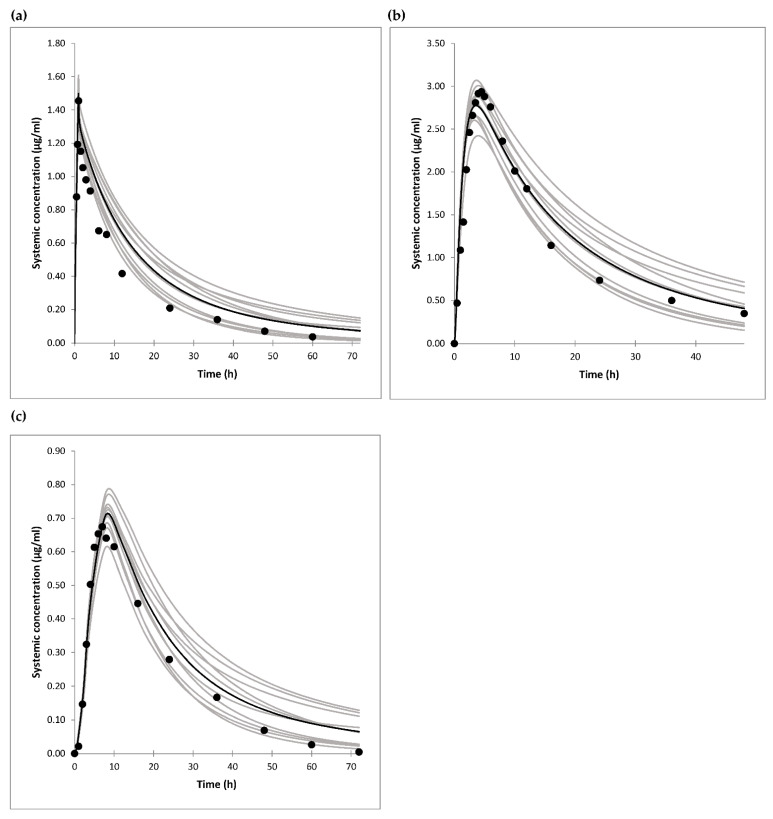
(**a**) Observed [35] (circles) and simulated (lines) plasma concentration-time profiles following 30 mg gliclazide i.v. infusions. (**b**) Observed [44] (circles) and simulated (lines) plasma concentration-time profiles following single oral administration of 80 mg gliclazide IR tablets. (**c**) Observed [35] (circles) and simulated (lines) plasma concentration-time profiles following single oral administration of 30 mg gliclazide modified-release (MR) tablets. The grey lines represent means of the ten simulated trials, and the black line represents the mean of the whole virtual population.

Simulations were performed to match the protocol and conditions from clinical trials in Caucasians reported in the literature. The concentration-time profiles following 30 mg gliclazide i.v. infusion [35], single oral administration of gliclazide 80 mg IR tablet [44], and single oral administration of gliclazide 30 mg MR tablet [35], are shown in Figure 2.

The simulated PK profiles were in good agreement with published clinical data. The predicted C_max_, t_max_, and AUC values were within 0.80–1.38-fold of the observed data (Table 5).

### 3.3. Interaction between Omeprazole and Gliclazide—Clinical Trial and Simulations

**Figure 3:**
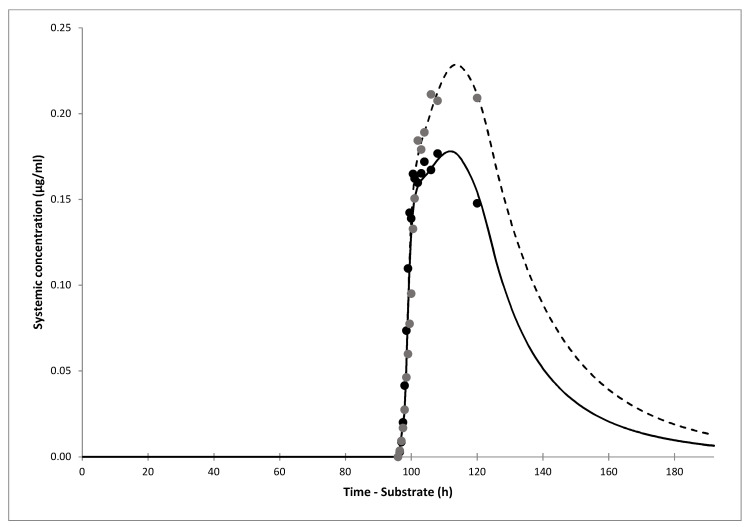
Observed (current trial, circles) and simulated (lines) plasma concentration-time profiles of gliclazide following oral administration of 40 mg gliclazide (half of 80 mg Diprian^®^ tablet) with placebo (black circles, solid line) or with 20 mg omeprazole (grey circles, dashed line). Lines represent the mean values for the whole virtual population. The mean values of the individual virtual trials were omitted for clarity.

In our clinical trial, 14 out of 15 volunteers (men, mean age 22.6 ± 2.7 years, mean body weight 79.8 ± 11.3 kg) completed both study periods. One volunteer was dropped from the analysis as he only completed one study period. There were no cases of hypoglycemia or any other adverse effect. The volunteers received half of the 80 mg Diprian^®^ tablet and were followed for only 24 h as the drug was registered as IR formulation in B&H. However, the concentration-time profiles showed that gliclazide was not eliminated from the body within 24 h and usual PK parameters including C_max_, t_max_, t_1/2_, and AUC_0–∞_ could not be determined (Figure 3, observed data). As obtained PK profiles resembled those seen with the MR formulations, we performed dissolution tests and confirmed the MR dissolution profile of Diprian. The experimentally obtained dissolution profile (Supplementary Figure S2a) was used as input data to simulate bioperformance of Diprian^®^ tablets. There was no difference in the initial gliclazide release rate between media pH 1.6 and pH 3.0. The mean cumulative amount of gliclazide dissolved after 26 h from Diaprel^®^ tablets was 89%, whereas only 37% of gliclazide was dissolved from Diprian^®^ tablets (Supplementary Figure S2).

Still, the observed PK profiles up to 24 h showed higher mean gliclazide AUC_0–24_ in the omeprazole phase compared to the placebo phase. The geometric mean ratio and 90% CI for gliclazide AUC_0–24_ between omeprazole and placebo phase was 1.13 (0.86–1.48), with upper confidence limit above the usual 1.25 boundary.

The observed and simulated PK profiles following co-administration of 40 mg gliclazide (the half of 80 mg Diprian^®^ tablet) with placebo or omeprazole are shown in Figure 3. The simulated profiles of gliclazide in both placebo and omeprazole phase captured well the observed data collected up to 24 h, with the predicted AUC_0–24_ within 1.0 and 1.1-fold of the observed data, respectively (Table 5). The simulated DDI C_max_ and AUC ratios for the current trial, as well as different CYP2C19 phenotypes, are shown in Table 6.

The predicted DDI AUC ratio for the current in vivo study was 1.40. If gliclazide IR tablets with complete absorption had been administered, the AUC ratio would have been 1.50, according to the model. The highest AUC ratio was predicted for NM phenotype, whereas for PM individuals no interaction was observed.

**Figure 4:**
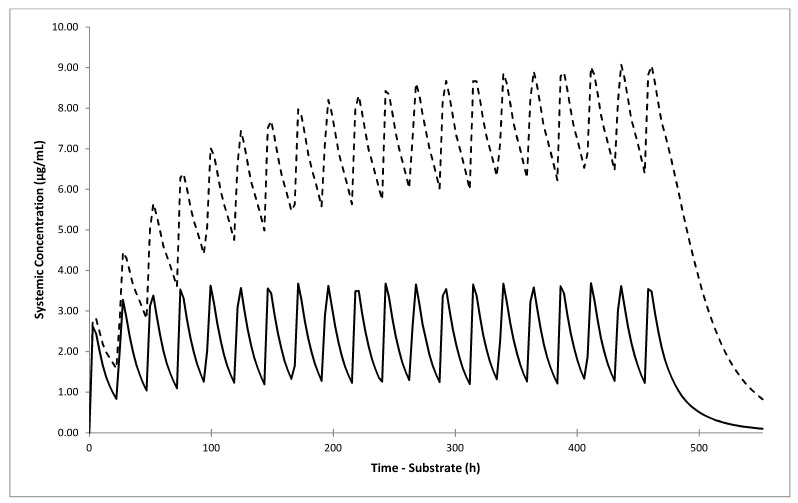
Simulated plasma concentration-time profiles of gliclazide following oral administration of 80 mg gliclazide IR tablet once daily alone (solid line) or concomitantly with 40 mg omeprazole twice daily (dashed line) in NM/RM/UM individuals for 20 days. Lines represent the mean values for the whole virtual population. The mean values of the individual virtual trials were omitted for clarity.

In the next step, we simulated four clinical scenarios of concomitant treatment of 80 mg gliclazide IR tablet once daily and 40 mg omeprazole once or twice daily in NM/RM/UM and IM individuals for 20 days. The simulated DDI C_max_ and AUC geometric mean ratios (90% CIs) are shown in Table 7. The predicted AUC increased 2.1 and 2.5-fold in IM individuals, and 2.6 and 3.8-fold in NM/RM/UM group, after 20 days of concomitant gliclazide administration with 40 mg omeprazole once and twice daily, respectively. The simulated concentration-time profiles of gliclazide during co-administration with 40 mg omeprazole twice daily in NM/RM/UM individuals are shown in Figure 4.

### 3.4. Omeprazole Treatment and the Risk of Severe Gliclazide-Induced Hypoglycemia in Patients with T2D 

The concomitant treatment with PPIs was not associated with increased odds of severe hypoglycemia in the whole group of T2D patients treated with gliclazide (173 patients and 826 controls), odds ratio (OR) (95% CI): OR = 1.33 (0.88–2.00), *p* = 0.173. Similar results were obtained for omeprazole, which was the most commonly used PPI drug in the cohort [OR = 1.31 (0.85–2.03), *p* = 0.221]. The loss-of-function CYP2C19*2 allele was not associated with hypoglycemia in the cohort with available genetic data (48 cases and 288 controls), OR = 0.50 (0.18–1.40), *p* = 0.187.

**Figure 5:**
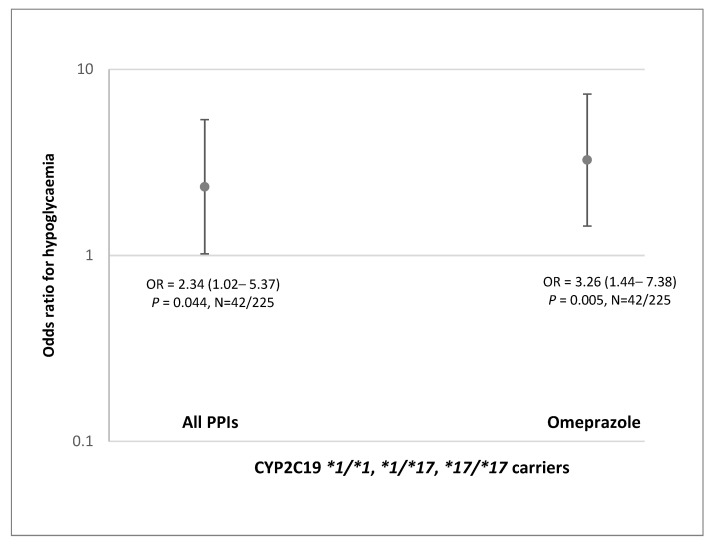
Effect of co-treatment with PPIs and omeprazole on gliclazide-induced hypoglycemia in T2D patients with CYP2C19 NM/RM/UM phenotype. Data are shown as odds ratios (OR) with 95% CIs. N, number of cases with hypoglycemia and controls.

However, when stratified by the *CYP2C19* phenotypes, the co-treatment with PPIs was associated with severe hypoglycemia only in CYP2C19*2 non-carriers [OR = 2.34 (1.02–5.37), *p* = 0.044] (Figure 5). This effect was mostly driven by omeprazole, which increased the odds of severe hypoglycemia over three-fold in patients with CYP2C19 NM/RM/UM phenotype treated with gliclazide [OR = 3.26 (1.44–7.38), *p* = 0.005] (Figure 5).

## 4. Discussion

This is the first study that explored the possible interaction between omeprazole and gliclazide. We used PBPK modeling approach to predict the effects of omeprazole on gliclazide PK and validated developed models using data from the clinical trial in healthy volunteers. In addition, we explored the influence of omeprazole on risk of severe hypoglycemia in real-world patients with T2D treated with gliclazide. Our results collectively suggest that omeprazole can increase exposure to gliclazide and thus increase the risk of side effects in individuals treated concomitantly with these two drugs if CYP2C19 enzyme is functional.

We first developed a PBPK model for omeprazole. Although Simcyp has a built-in model for omeprazole and few PBPK models for omeprazole were published earlier [27,28,29,42], the models did not perform well in the current version of Simcyp. Among others, one of the reasons is different and updated liver abundances of the enzymes. Considering that we were exploring interaction via polymorphic CYP2C19, we updated the phenotype frequencies of CYP2C9 and CYP2C19 enzymes and changed their abundancies in the liver according to the estimated enzyme activity scores for each phenotype. As the intrinsic clearance (Cl_int_) values for enzymes depend on their abundancies, they were back calculated based on in vivo clearance data. Although it is better to use in vivo clearance from i.v. administration in the retrograde model for Cl_int_ calculations, we used oral clearance value as it was reported for subjects with known CYP2C19 phenotype (NM/RM/UM phenotype), and gut metabolism of omeprazole is expected to be negligible [43]. The non-linear PK of omeprazole was previously best captured by published models for omeprazole enantiomers [28]. We managed to optimise values for parameters related to mechanism-based inhibition of CYP2C19 by omeprazole (K_app_ and k_inact_) using published data for enantiomers as starting values [28]. The simulation of changes in the amount of active CYP2C19 following 5-day dosing with 40 mg omeprazole once daily showed that the lowest level of active CYP2C19 was 11% of baseline, in line with the predicted lowest level of 14% obtained in a previous study based on enantiomers [28]. The model simulated well four drug plasma concentration-time profiles, as well as C_max_ and AUC values following single and multiple oral administration of 20 mg and 40 mg omeprazole. All predicted PK parameters were within 1.1-fold of the clinical data.

The model for gliclazide was developed using in vitro, in silico and in vivo data as inputs. As gliclazide is a substrate of liver OATP1B1 transporter [37], which can contribute to its liver uptake and hence the metabolism and elimination, the Cl_int_ values for enzyme kinetics could not be back calculated from the in vivo data. Only one in vitro study of gliclazide metabolism was published using human liver microsomes and a panel of recombinant human CYPs [10]. Based on the total microsomal intrinsic clearance, and the percentage of inhibition of metabolic pathways by CYP selective inhibitors, the contribution of CYP2C9 to gliclazide metabolism was estimated to be at least 65%, and CYP2C19 maximally 35% according to this study [10]. However, microsomes were prepared from six human livers which were not genotyped for CYP2C19 polymorphisms. Furthermore, later in vivo studies showed that gliclazide PK is affected mainly by CYP2C19 polymorphisms [11,12,55]. Based on these PK clinical studies in individuals with different CYP2C19 and CYP2C9 genotypes [11,12], it was estimated that the contribution of CYP2C9 to gliclazide metabolism is around 24%, and of CYP2C19 around 76% [36]. Taking this into account, the Cl_int_ values for enzymes were calculated based on the overall in vitro microsomal intrinsic clearance [10], and f_m_ values estimated from in vivo studies [36].

By using enzyme Cl_int_ values only, clearance of the drug after i.v. administration was largely underpredicted. Therefore, we extended our model to include in vitro obtained values for OATP1B1-mediated gliclazide transport [37], and optimized RAF value for the transporter to fit the observed data after i.v. drug administration [35], as well as after oral drug dosing [35,44]. Namely, only one study describing gliclazide PK after i.v. administration was found in the literature and referred to i.v. infusion [35], so oral drug dosing studies were also taken into account. The RAF or relative expression factor (REF) frequently needs to be optimized in the PBPK models due to a difference in expression of the transporter between liver and the transfected cell line. The RAF value of 5.75 was in line with bottom-up derived REF values of 5.9 and 8.7 for different OATP1B1 substrates [56] based on measured OATP1B1 expression in the liver [57], isolated hepatocytes [57], and transfected HEK-293 cells in other studies [56].

Another parameter that had to be optimized was MRP2-mediated intrinsic clearance as t_max_ values for oral formulations were underpredicted in the preliminary simulations. It has been shown that gliclazide is a substrate for gut Mrp2 and Mrp3 transporters in rats [41], but in vitro and human data are lacking. As intestinal basolateral transporters are not yet included in the Simcyp ADAM model, only apical transporter MRP2 was added to the model. After this adjustment, the simulated t_max_ values were in accordance with the observed data after oral drug dosing.

The simulations of gliclazide PK showed good agreement with clinical data after i.v. infusion, and oral administration of gliclazide IR and MR tablets. The model showed that only 30% (19–43%) of gliclazide was absorbed in our current clinical trial. The simulation of the interaction showed that the predicted DDI AUC ratio of gliclazide between omeprazole and placebo phases was 1.40 (1.35–1.45). The predicted ratio would have been 1.50 (1.44–1.56) if IR tablets with complete absorption were administered. The predicted DDI C_max_ ratio for the current trial was 1.26 (1.23–1.29), however, C_max_ would not have been impacted significantly by omeprazole dosing if gliclazide IR tablets were administered, according to the model.

The simulated DDI AUC ratio after 5-day dosing with 20 mg omeprazole was 1.55, 1.49, 1.40, and 1.01 for NM, RM/UM, IM and PM individuals, respectively. A slightly lower AUC ratio in RM/UM compared with NM individuals was due to lower predicted omeprazole systemic and liver concentrations and thus weaker inhibitory effect on CYP2C19. The lowest simulated percentage of active CYP2C19 was 31% in RM/UM compared to 21% in NM individuals. On the other hand, AUC ratio of 1.40 in IM individuals was comparable to the ratios obtained in NM and RM/UM phenotypes. Although simulated CYP2C19 f_m_ value for gliclazide in IM individuals was only 59%, the interaction effect was similar to the one in the NM/RM/UM group, due to higher predicted systemic concentrations of omeprazole. As expected, no interaction effect in PM subjects was found due to low or absent CYP2C19.

Although the predicted omeprazole-gliclazide interaction effect was not large, when we simulated clinical scenarios of concomitant multiple dosing of both drugs for 20 days in NM/RM/UM individuals, the DDI AUC ratio increased to 2.57 (2.39–2.76) if 40 mg omeprazole was administered once daily, and to 3.80 (3.48–4.15) if it was administered twice daily. The predicted DDI AUC ratio in IM individuals also increased to 2.13 (2.00–2.27) and 2.53 (2.35–2.73) after co-administration of 40 mg omeprazole once and twice daily, respectively.

We used the Simcyp population of healthy volunteers for all simulations, as there is no special built-in population of patients with T2D in the Simcyp. However, considering the older age of these patients and possible age-related PK changes such as limited drug clearance due to decreased liver blood flow and reduced kidney function, as well as frequent concomitant therapy with other interacting drugs, the concentrations of drugs and thus interaction effect can be greater in older patients compared to young healthy volunteers [58]. Interestingly, a recent study found lower mean metabolic activities of CYP2C19, CYP2B6, and CYP3A enzymes in patients with T2D compared to non-T2D individuals [59]. However, although lower activity of CYP2C19 can decrease the CYP2C19 f_m_ value for gliclazide, the inhibition of CYP21C9 could be stronger due to higher systemic concentrations of omeprazole. In line with this, in a study that explored the effect of ageing on omeprazole PK, the omeprazole AUC was higher in elderly compared to young Japanese volunteers following single i.v. dose of omeprazole [60].

Finally, we explored the effect of co-treatment with omeprazole and other PPI drugs in real-world patients with T2D treated with gliclazide. Although patient cohort with available genetic data was not large, we showed that patients with CYP2C19 NM/RM/UM phenotype treated with omeprazole had 3.26-fold (1.44–7.38) higher odds of severe hypoglycemia. These results corroborate higher systemic exposure to gliclazide predicted for NM/RM/UM individuals. No effect was seen in a very small group of patients carrying CYP2C19*2 allele.

To our best knowledge, there have been no studies exploring potential interaction between any PPI and gliclazide. Data regarding potential DDI between PPIs and other SUs in humans are also limited. One study conducted in healthy volunteers showed no effect of pantoprazole on glibenclamide PK [61]. As glibenclamide is metabolized by CYP2C9 and CYP3A4, and other SUs, including glimepiride, glipizide and gliquidone, are metabolized mostly by CYP2C9 [62], potential PK interactions with PPIs are not expected. On the other hand, other PPIs could also interact with gliclazide via CYP2C19, however, except for esomeprazole, their potential for DDIs seems to be weaker [15].

The main limitation of our study are incomplete in vivo gliclazide PK profiles in omeprazole and placebo phase obtained in the current clinical trial. After we informed the Agency for Medicines and Medical Devices of Bosnia and Herzegovina about the unexpected MR profile of Diprian^®^ tablets, the drug was withdrawn from the market and thus patients in B&H stopped receiving this possibly inefficient drug. Still, we had collected data up to 24 h, when drug elimination had mostly prevailed, and PK profiles in both phases were well simulated. Our PBPK models confirmed the mechanistic hypothesis of the interaction between the two drugs via polymorphic CYP2C19 enzyme and also allowed us to predict different clinical scenarios.

Our results have potential clinical relevance. The predicted increase in gliclazide AUC after 20-day of concomitant dosing with omeprazole shows that NM/RM/UM individuals may have almost 4-times higher exposure to gliclazide. This is in line with over 3-fold higher odds of hypoglycemia seen in NM/RM/UM patients with T2D treated concomitantly with omeprazole and gliclazide. The predicted AUC increase in IM individuals was lower but still over 2-fold. This may lead to an increased risk of gliclazide side effects in all patients treated with omeprazole, except those with the CYP2C19 PM phenotype. The risk may be even higher if other clinical risk factors for hypoglycemia are present. Avoiding co-prescribing omeprazole in these patients may prevent serious consequences of severe hypoglycemia induced by gliclazide treatment.

In conclusion, we applied PBPK models to simulate the clinical PK profiles and to assess the potential interaction between omeprazole and gliclazide. Developed models predicted 1.4–1.6-fold higher gliclazide AUC in all CYP2C19 phenotypes except PM, after 5-day treatment with 20 mg omeprazole. The predicted gliclazide AUC increased 2.1 and 2.5-fold in IM individuals, and 2.6 and 3.8-fold in the NM/RM/UM group after 20 days of concomitant gliclazide administration with 40 mg omeprazole once and twice daily, respectively. The predicted results were corroborated by findings in patients with T2D which demonstrated over 3-fold higher odds of severe gliclazide-induced hypoglycemia in patients with CYP2C19 NM/RM/UM phenotype treated with omeprazole. These results indicate that omeprazole may increase the risk of hypoglycemia in the majority of patients treated with gliclazide. Further studies are needed to confirm this previously unrecognized but potentially serious DDI and drug–drug–gene interaction.

## Figures and Tables

**Table 3:** Dissolution test conditions.

Represented GI Region	Stomach	Duodenum and Jejunum	Ileum	Colon
Conditions without omeprazole *				
pH value/Volume (mL)/Residence time (h)	1.6 (a)/300/0.5	5.8 (b)/900/2	6.8 (c)/900/1.5	6.0 (d)/900/22
Conditions with omeprazole *				
pH value/Volume (mL)/Residence time (h)	3.0 (e)/300/0.5	5.8 (f)/900/2	6.8 (c)/900/1.5	6.0 (d)/900/22

* Conditions simulating gastric pH effects of omeprazole use; GI, gastrointestinal; (a) 0.03 M HCl solution; (b) the pH adjusted by the addition of phosphate buffer (225 mL 0.15 M KH_2_PO_4_, 75 mL 0.15 M K_2_HPO_4_ and 300 mL H_2_O); (c) for media pH 6.8, the pH adjusted by replacing the withdrawn media aliquot with an equal volume of 21 mL 1 M KOH solution; (d) the pH adjusted by the addition of 6 mL 3 M HCl; (e) 0.03 M HCl solution adjusted to pH 3.0 by adding KOH pellets; (f) pH adjusted by the addition of phosphate buffer (270 mL 0.3 M KH_2_PO_4_, 30 mL 0.3 M K_2_HPO_4_ and 300 mL H_2_O).

**Table 4:** Comparison of PK parameters between the simulated and observed data for omeprazole.

Dosing	PK Parameter	Observed	Simulated	Fold Error Simulated/Observed
20 mg day 1	C_max_ (µM)	1.04 (0.75–1.44)	1.10 (0.98–1.23)	1.06
	AUC_0–∞_ (µM × h)	1.04 (0.64–1.72)	0.97 (0.81–1.17)	0.93
20 mg day 5	C_max_ (µM)	1.43 (1.02–2.00)	1.52 (1.35–1.72)	1.06
	AUC_0–∞_ (µM × h)	1.63 (0.96–2.78)	1.67 (1.36–2.05)	1.02
40 mg day 1	C_max_ (µM)	2.32 (1.71–3.16)	2.63 (2.34–2.95)	1.13
	AUC_0–∞_ (µM × h)	2.44 (1.53–3.91)	2.79 (2.30–3.38)	1.14
40 mg day 5	C_max_ (µM)	3.87 (2.99–5.02)	4.00 (3.58–4.48)	1.03
	AUC_0–∞_ (µM × h)	5.79 (3.60–9.33)	5.57 (4.56–6.79)	0.96

Data are shown as geometric means with 95% CIs. Observed data are from Hassan-Alin et al., 2005 [30].

**Table 5:** Comparison of PK parameters between simulated and observed data for gliclazide.

Dosing (Study)	PK Parameter	Observed	Simulated	Fold Error Simulated/Observed
i.v. infusion 30 mg [35]	C_max_ (µg/mL)	1.39 (1.16–1.67)	1.54 (1.51–1.58)	1.11
	AUC_0–72_ (µgh/mL)	15.5 (13.0–18.5)	21.4 (19.1–24.0)	1.38
	AUC_0–∞_ (µgh/mL)	17.2 (14.5–20.3)	22.9 (19.9–26.2)	1.33
80 mg IR tablet [44]	C_max_ (µg/mL)	3.51 (3.24–3.80)	2.81 (2.70–2.91)	0.80
	t_max_ (h)	3.66 (3.10–4.33)	3.54 (3.35–3.73)	0.97
	AUC_0–48_ (µgh/mL)	45.5 (38.9–53.3)	51.0 (45.8–56.9)	1.12
	AUC_0–∞_ (µgh/mL)	50.9 (42.8–60.5)	59.1 (51.1–68.4)	1.16
30 mg MR tablet [35]	C_max_ (µg/mL)	0.71 (0.62–0.82)	0.70 (0.66–0.74)	0.99
	t_max_ (h) *	7 (4–10)	8.4 (5.1–13.5)	1.20
	AUC_0–72_ (µgh/mL)	14.4 (12.4–16.8)	15.7 (13.9–17.8)	1.09
	AUC_0–∞_ (µgh/mL)	15.6 (13.5–18.1)	17.0 (14.7–19.7)	1.09
40 mg tablet (current trial, placebo phase)	C_max_ (µg/mL)	-	0.18 (0.17–0.19)	-
	t_max_ (h) *	-	15.4 (4.4–26.8)	-
	AUC_0–24_ (µgh/mL)	3.29 (2.65–4.09)	3.33 (3.09–3.59)	1.01
	AUC_0–96_ (µgh/mL)	-	5.51 (4.89–6.21)	-
	AUC_0–∞_ (µgh/mL)	-	5.61 (4.96–6.36)	-
40 mg tablet (current trial, omeprazole phase)	C_max_ (µg/mL)	-	0.23 (0.21–0.25)	-
	t_max_ (h) *	-	17.7 (4.5–27.2)	-
	AUC_0–24_ (µgh/mL)	3.73 (2.81–4.93)	4.09 (3.78–4.42)	1.10
	AUC_0–96_ (µgh/mL)	-	7.65 (6.72–8.71)	-
	AUC_0–∞_ (µgh/mL)	-	7.86 (6.86–9.00)	-

Data are shown as geometric means with 95% confidence intervals (CIs), or as * medians (minimum–maximum).

**Table 6:** Simulated DDI C_max_ and AUC ratios for interaction between omeprazole and gliclazide.

Population	Gliclazide Dosing	C_max_ Ratio	AUC Ratio
Current in vivo trial (NM/RM/UM)	40 mg Diprian^®^ tablet	1.26 (1.23–1.29)	1.40 (1.35–1.45)
NM/RM/UM	40 mg IR tablet	1.09 (1.08–1.10)	1.50 (1.44–1.56)
NM	80 mg IR tablet	1.10 (1.09–1.11)	1.55 (1.49–1.60)
RM/UM	80 mg IR tablet	1.10 (1.09–1.11)	1.49 (1.43–1.55)
IM	80 mg IR tablet	1.06 (1.06–1.07)	1.40 (1.36–1.43)
PM	80 mg IR tablet	1.00 (1.00–1.00)	1.01 (1.01–1.01)

Omeprazole (20 mg) was administered for 5 days, and a single dose of gliclazide was administered on day 5. DDI C_max_ and AUC_0–∞_ geometric mean ratios are shown with 90% CIs. NM, normal metabolizers; RM, rapid metabolizers; UM, ultrarapid metabolizers; IM, intermediate metabolizers; PM, poor metabolizers.

**Table 7:** Simulated DDI C_max_ and AUC ratios for interaction between omeprazole and gliclazide after 20 days of concomitant dosing.

Population	Omeprazole Dosing	C_max_ Ratio	AUC Ratio
NM/RM/UM	40 mg q.d.	1.67 (1.60–1.75)	2.57 (2.39–2.76)
NM/RM/UM	40 mg b.i.d.	2.11 (1.99–2.25)	3.80 (3.48–4.15)
IM	40 mg q.d.	1.60 (1.53–1.67)	2.13 (2.00–2.27)
IM	40 mg b.i.d.	1.78 (1.69–1.88)	2.53 (2.35–2.73)

Gliclazide (80 mg IR tablet) and omeprazole were administered concomitantly for 20 days. DDI C_max_ and AUC_0–96_ ratios on day 20 are shown as geometric mean ratios with 90% CIs. NM, normal metabolizers; RM, rapid metabolizers; UM, ultrarapid metabolizers; IM, intermediate metabolizers; q.d., once daily; b.i.d., twice daily.

## Data Availability

The data presented in this study are available on request from the corresponding author.

## Supplementary Materials

The following are available online at https://www.mdpi.com/article/10.3390/jpm11050367/s1, Figure S1: Simulation of the amount of active CYP2C19 during 5-day dosing with 40 mg of omeprazole once daily, Figure S2: Mean dissolution profiles of the half of the 80 mg Diprian^®^ tablets (a) and 60 mg Diaprel^®^ MR tablets (b).
